# A qualitative assessment of perceived barriers to effective therapeutic communication among nurses and patients

**DOI:** 10.1186/s12912-019-0328-0

**Published:** 2019-02-11

**Authors:** Vida Maame Kissiwaa Amoah, Reindolf Anokye, Dorothy Serwaa Boakye, Enoch Acheampong, Amy Budu-Ainooson, Emelia Okyere, Gifty Kumi-Boateng, Cynthia Yeboah, Jennifer Owusu Afriyie

**Affiliations:** 1 Centre for Disability and Rehabilitation Studies, Department of Community Health, School of Medical Sciences, Kwame Nkrumah University of Science and Technology, Kumasi, Ghana; 2 Department of Nursing, Garden City University College, Kumasi, Ghana; 3 School of Public Health, Department of Health Education and Promotion, Kwame Nkrumah University of Science and Technology, Kumasi, Ghana

**Keywords:** Barriers, Effective, Therapeutic communication, Nurses, Patients

## Abstract

**Background:**

Therapeutic communication is essential in the provision of quality healthcare to patients. The purpose of this study was to explore the perceived barriers to effective therapeutic communication among patients and nurses at Komfo Anokye Teaching Hospital,Kumasi.

**Methods:**

An exploratory study design was employed using a qualitative approach. A purposive sampling technique was used to select 13 nurses and patients who were interviewed using an unstructured interview guide. Interviews were audio-taped, transcribed verbatim and analyzed using thematic content analysis.

**Results:**

Patient-related characteristics that were identified as barriers to effective therapeutic communication included socio-demographic characteristics, patient-nurse relationship, language, misconception, as well as pain. Nurse-related characteristics such as lack of knowledge, all-knowing attitude, work overload and dissatisfaction were also identified as barriers to effective therapeutic and environmental-related issues such as noisy environment, new to the hospital environment as well as unconducive environment were identified as barriers to effective therapeutic communication among patients and nurses at Komfo Anokye Teaching Hospital,Kumasi.

**Conclusion:**

Nurse-patient communication is an inseparable part of the patients’ care in every health setting; it is one of the factors that determine the quality of care. Several patient-related characteristics, nurse- related characteristics and environmental-related issues pose as barriers to effective therapeutic communication at Komfo Anokye Teaching Hospital,Kumasi and have ultimately; resulted in reducing effective communication at the wards. Therefore, all the barriers must be eradicated to promote effective therapeutic communication.

## Background

Therapeutic communication is essential in the provision of quality healthcare to patients. According to the American Nurses Association [1], nurses serve the role as patient advocates and must, therefore, preserve a therapeutic and professional nurse-patient relationship in their professional role with specific boundaries to their role. This makes it necessary that nurses adopt techniques in interacting with patients within the clinical setting which is an important part of their work in the provision of healthcare to patients.

Therapeutic communication involves a direct face to face contact with patients that focus on enhancing the physical and emotional well-being of patients [2]. A variety of techniques are used by nurses in communicating with patients. In therapeutic communication, there is a verbal and non-verbal flow of information between nurses and patients [3]. The verbal aspect of communication employs the use of words whilst non-verbal communication makes use of non-verbal cues such as eye contact, body language, and facial expression [3].

Bournes and Mitchell [4] state, “Health is the way people go on and live what is important to them, moment to moment and day to day”. The recognition of effective therapeutic communication as a critical part of healthcare provision has been highlighted in several studies [5]. Therapeutic communication has the potency of increasing patients’ knowledge and understanding, enhancing trust and self-health skills, increase adherence, providing comfort and facilitating the management of emotions key to patients’ health and well-being [2]. Again, it has been well documented in several studies that medical practice is affected with the quality of communication between patients and clinicians as more medical errors occur with less effective communication within the clinical setting [6, 7].

Nevertheless, several factors such as the environment/surroundings, circumstances and timing affect the restorative and soothing facets of patients that give significance to therapeutic communication [8]. For instance, in emergency cases where there will be little time for verbal interaction, the use of non-verbal cues such as the holding of hand could carry much more words to patients [8]. Even in cases where nurses are much experienced in therapeutic communication, there can still be a gap in communication as sometimes it becomes difficult to understand patients from their own viewpoints [8].

Therefore, this study sought to investigate the barriers to effective therapeutic communication among patients and nurses at the Komfo Anokye Teaching Hospital in Kumasi.

## Methods

### Study site

Komfo Anokye Teaching Hospital (KATH) is a tertiary health facility premise in the environs of Kumasi within the Ashanti Region and ranked as the second largest hospital in Ghana. Komfo Anokye Teaching Hospital (KATH) has a bed capacity of about 1000 with twelve (12) clinical directorates providing healthcare services to the people within the region as well as handling referrals from other closer regions.

### Study design

The study employed an exploratory based design which followed a qualitative approach to investigate Nurses’ and Patients’ experiences and views on the barriers to effective therapeutic communication to serve as a springboard for further studies.

### Study population

The study population included patients and nurses at Komfo Anokye Teaching Hospital.

### Inclusion criteria

Participants included in the study were individuals who had been admitted for a minimum of 3 to 4 days. This meant that participants would have communicated regularly with the nurses during their stay. Registered nurses employed full-time and having worked for four months or more at KATH were also included.

### Exclusion criteria

Unconscious and patients who had not been on admission for up to a minimum of 3 days were excluded from the study. Nurses who were not full time and had not worked for more than 4 months were also excluded.

### Techniques, instruments for data collection and analysis

An in-depth interview guide was used as the data collection instrument to gather in-depth information from participants. The interview guide allowed the researcher to probe further in order to understand and explore participant contributions in as much depth as possible. An unstructured interview guide which followed an open-ended approach permitted the in-depth investigation of experiences and views regarding nurse-patient communication. The interview guide contained interview questions on the demographic profile of nurses and patients as well as interview questions on Nurse-related barriers; Patient-related barriers and Environmental-related barriers to Therapeutic Communication.

The interview days and time were discussed with participants and each interview was scheduled at their convenience. Interviews were then audio-taped so that participants’ responses could later be transcribed verbatim. The researcher used two weeks for data collection (3rd March to 17th March 2016) with each interview lasting approximately 45 to 60 min. The collection of data was done by two of the authors (sixth and seventh authors) and assisted by two (2) research assistants. Participants were informed about the time before commencement. The data collected was then transcribed verbatim and analyzed through thematic content analysis. This was done by listening to tape recordings and transcribing the content. The transcript was coded by going through the transcript line by line and paragraph by paragraph, to find significant statements and codes according to the topics addressed. The similarities and contrast within the data were compared by the investigators and data that seemed to cluster together were sorted into categories.

### Sampling and sampling techniques

The participants were purposively selected to participate in the study based on the characteristics they exhibited which were of interest to the researchers and were able to provide the needed information. According to Patton [9], the logic and power of purposeful sampling lie in selecting information-rich cases for in-depth study. A sample of 13 participants was used for the study which was made up of 6 nurses and 7 patients. The interviews were conducted till a saturation point was reached after interviewing the 13th participant. Saturation refers to the point at which new data collected and analyzed does not provide further meaning to the research question [10].

### Validity and reliability

To ensure rigour, or the integrity in which the study was conducted, and ensure the credibility of findings in relation to qualitative research, several steps were taken to enhance the validity and reliability of the study [11].

Firstly, comparisons were done to find out similarities and differences across accounts to ensure that all different perspectives were represented.

Also, active and continuous reflection was done by the researcher during the interpretation of data to ensure the quality of the data and to mirror participants’ experiences to add credibility to the study. The context of the research and assumptions central to the study were thoroughly described to achieve transferability. The criteria applied were made explicit, according to the purpose and orientation of the study [12].

Furthermore, other researchers were engaged to reduce research bias and the researchers and supervisor again ensured that the findings, conclusions, and recommendations were braced by the data collected and that the interpretation of the data was meaningful and relevant to the study.

### Limitations of the study

The study was conducted at Komfo Anokye Teaching Hospital which is a single facility and therefore the findings cannot be generalized. Some of the participants were not willing to respond to some of the interview questions due to its sensitive nature. However, these limitations did not influence the findings of this study in any negative way.

## Results

Thematic content analysis was used to analyze data collected based on the aims of the study. The results included background characteristics of study participants as well as the main and sub-themes of the study. Three main themes were derived from the data collected. The themes included; patient-related barriers with sub-themes personal/ social characteristics; patient-nurse relationship; language barriers as well as misconception and pain. The nurse related barriers came with sub-themes such as availability of nurses; inadequate knowledge; all-knowing attitude; dissatisfaction as well as the disease state and family interference. The environmental barriers included sub-themes such as noisy environment; new to the hospital environment and unconducive environment.

### Background characteristics of study participants

Table 1 shows the background characteristics of the study participants.From the table,five (5) of the study participants were females whiles eight (8) were males. The participants were between the ages of 20 and 50 years and most of them were between the ages of 20 and 30 years. Moreover, six of the participants were married and seven were single. Most of the patient participants have attained their tertiary education while most of the nurses have attained their degree in nursing.

**Table 1:** Background characteristics of both patients and nurses

Variable	Frequency	Percentage
NURSES		
Sex		
Male	4	67
Female	2	33
Age		
20–30	3	50
31–40	2	33
40 and above	1	17
Marital Status		
Married	4	67
Single	2	33
Educational Level		
Diploma	1	17
Degree	5	83
Duration of service (years)		
1–3	4	67
3 and above	2	33
PATIENTS		
Sex		
Male	4	57
Female	3	43
Age		
20–30	4	57
31–40	2	29
41 and above	1	14
Marital Status		
Married	3	43
Single	4	57
Educational Level		
Primary	1	14
S.H.S	2	29
Tertiary	4	57

### Main findings

#### Patient-related barriers

Patient-related barriers are those obstacles directly from patients that inhibit effective therapeutic communication. Sub-themes that emerged are personal/social characteristics, patient-nurse relationship, language barriers, misconception, and pain.

#### Personal / social characteristics

These included characteristics such as age, religion, ethnicity among others that have the tendency of influencing communication**.**

One Participant revealed that;

*Once they age, conditions such as dementia sets in and this causes their level of interpretation and understanding to go down and it becomes difficult communicating with them. Those at the extremes of age will have difficulty as compared to those who are in their middle ages. Sometimes the younger ones act like they understand what we tell them and they are okay but in actual sense, they do not understand (Participant 2).*

One nurse also reported that religious beliefs and culture play a key role;

*Some patients are Muslims and would not want females to attend to them but they prefer males. People from different parts of Ghana have different cultures thus, culture and religion, patient status, all do count to add up to the personal barriers. There are also patients who would say no to blood but as a nurse, you have to use your discretion and this can alter effective communication between you and the patient (Participant 13).*

A greater number of the nurses admitted that the cultural background of patients affected their communications. One echoed;

*Sometimes the culture of some people will require you to bow whenever you attend to or meet them but as a nurse, you work with limited time and therefore cannot be bowing to everyone you meet and this may portray you the nurse as been insolent thus, affecting the level of communication (Participant 11).*

### Patient-nurse relationship

Patient-Nurse Relationship is essential for effective healthcare delivery. In this study, patients complained about their relationship with the nurses and the way the nurses attend to them when they are in need. The nurses also admitted that the kind of relationship between the client and them also influences the level of effective communication.

One patient admitted that;

*Our relationship is okay but I think their swiftness is a little questionable, they sit always but I think maybe it’s because they are busy but for me, their swiftness is not too good just not too good (Participant 1).*

### Misconception

Misconceptions can distort effective communication. One individual may perceive another to be of a certain trait, character or of a certain attitude.

Patient revealed that;

*I think we come with preconceived ideas because of what we hear about the nurses (Participant 2).*

*Some of us have misconceptions about nurses that they are rude and disrespectful so we already have something in mind before coming to the ward (Participant 3).*

### Pain

Pain is one thing that can change one’s mood and influence his/her behaviour. Participants verbalized how pain act as a barrier to effective therapeutic communication;

*Sometimes I don’t blame the patients, because the pain is too much for them to bear, they wouldn’t want to engage themselves in the conversation going on. In fact, whiles you are in pains and someone tries to even communicate with you, you sometimes get angry (Participant 2).*

Another participant had this to say:

*Because of the fact that the person is suffering and going through a lot when they call the nurses for one or two times and we don’t attend to them they lose their temper and begin to alter insults (Participant 5).*

### Language

Language can act as a barrier to any form of communication and effective therapeutic communication is not an exception. Some of the patients complained that nurses mostly resorted to the Twi language when most of the patients have difficulties in understanding Twi.

*I am a Voltarian and would like the nurses to speak English because I do not understand Twi but they always speak Twi. They should speak in languages that we will understand and I know every nurse can speak English so I do not know why they normally prefer to speak Twi whiles they know some of us cannot speak Twi well (Participant 3).*

*There was a patient here who was a northerner and could not communicate so whenever he needed something he had to wait for his relatives to come so that he will communicate his needs through them to the nurses (Participant 7).*

Ghana is an Anglophone speaking country and most residents don’t speak French. Therefore, if a patient cannot speak English, it will be difficult communicating. Occasionally, foreigners who speak French and other languages and are living in Ghana visit the Hospital. Majority of the nurses commented on how language was a problem to effective therapeutic communication. Nurses complained of having patients from different tribes and countries which makes it difficult for them to communicate effectively.

*There are people admitted here who speak French and as for me, I have never spoken French before so it makes it difficult for me to communicate with them. Also, because KATH is a referral point for many hospitals and clinics we admit people from mostly Nigeria and China and its quite difficult talking to those who cannot speak English, sometimes we have to resort to sign language and even that we are not good in it. There was an occassion when we had a patient from Upper West who couldn’t speak Twi so we had to resort to sign language (Participant 12).*

### Nurse-related barriers

This category includes barriers related to attributes of the nurse. These attributes can be barriers in establishing a therapeutic nurse-patient relationship in the hospital. Six sub-themes emerged from it and they are inadequate knowledge, disease state, availability of nurses, all-knowing attitude, family interference, and dissatisfaction.

### Availability of nurses

The Nurses complained that due to the small number of nurses and the workload it becomes difficult attending to all patients as and when they call.

One Nurse revealed;

*Workload has been a factor, when nurses have a lot of work to do they will not have time to explain things to patients they will tell the patient ‘don’t you know I am very busy, don’t you know I am overburden’ especially with women whose threshold of managing stress is low as compared to men so that is a point (Participant 11).*

*Ooh! because there are few nurses at the ward sometimes you would want a nurse to attend to you but he or she might be working on another patient so in such case the nurse cannot divide him or herself into two to attend to you both. So you have to wait for quite some time and at times due to stress they end up forgetting that you called them (Participant10).*

A patient stressed that;

*One thing is that the nurses that are taking care of us are very few. So most at times the nurses here, lets say patients are 31 and only 4 nurses are taking care of us. Anytime you call the nurse, she will be busy doing something else and will tell you that she will be back soon. And as a human being, you can forget about things so easily. So as the nurse is attending to a sick patient, she may also come to your direction and another sick person will also call the nurse so hardly do we communicate with them as often. They are always busy (Participant 6).*

Another Nurse commented;

*Because there are few nurses at the ward, sometimes you would like a nurse to attend to you but he/she might be working on another patient (Participant 13).*

### Inadequate knowledge

Most nurses admitted and verbalized that, some nurses had little knowledge on how to communicate with others. Lack of knowledge on therapeutic communication on the part of some nurses also contributed to ineffective therapeutic communication. If there is a close relationship between the patient and the nurse, a patient can speak out all their problems to the nurse.

One of the Nurses echoed;

*I will say ignorance or nurse is not well abreast of what effective therapeutic communication is. A nurse who knows what effective therapeutic communication is will use it, especially if the nurse knows what it does to the healing process (Participant 10).*

### All-knowing attitude

Attitude refers to the predisposition to behave in a certain manner. Majority of the nurses verbalized that because some of the patients have stayed at the ward for a long time and in an era where most patients are educated, they think they know more about the nursing procedures and thus, do not adhere to whatever their health care provider says.

The nurses revealed;

*Patients who are learned tend to give a lot of instructions when performing any procedures on them. For example, when they are allergic to pethidine injection they assume every injection you give them is pethidine and will start complaining, this makes communication difficult (Participant 12).*

*You see when the patients stay on the ward for a very long time, they begin to act that they know everything you do for them. So for instance, when you are dressing their wounds, they go like do this do that trying to dictate or tell you what to do and if you resist they will claim you are all-knowing (Participant 11).*

### Dissatisfaction

When one is not pleased with a service provider or a process, it may distort effective communication between the individual and another person. Most of the nurses identified dissatisfaction with services provided by nurses  as the predominant barrier to effective communication.

One Nurse revealed;

*I would not say so; I wouldn’t say they are satisfied. Even when you get the time to talk to them the duration of the conversation is usually minimal okay, because of the work overload and the number of nurses to attend to patients you won’t get the time to communicate with our patients. But sometimes we do try but I will say that it is not the best (Participant 10).*

Another echoed;

*In terms of the level of satisfaction of patients, it may vary from one patient to another. Generally, I will give them 40% because I think they are not satisfied since we don’t explain things thoroughly to them. We don’t normally explain the condition and even the adverse effect of their drugs to them. All these things must be done by us and are not effectively done (Participant 11).*

### Disease state

All nurses verbalized that, the disease state and mental status of patients also affect the level of communication between nurses and patients.

One of the Nurses’ commented;

*When the patient is unconscious or just returned from surgery, it becomes difficult communicating with them and this can also reduce the quality of care provided to them (Participant 1)*.

Another Nurse also added;

*Currently we have a mental disoriented patient on the ward and in this case, communicating with such a patient is a problem which also leads to ineffective therapeutic communication (Participant 9).*

### Family interference

A family may interfere in a service process in order to influence outcomes. Another problem that the nurses admitted to facing is family interference in most of the procedures at the ward. Also, the kind of behaviour exhibited by clients’ families also affects how they communicate with them.

A Nurse said;

*Sometimes the patient may be very sick and may not need any relatives to be there, they need some rest and when you want to restrict them (relatives) there is trouble. Some want to even dictate to you. They want to plan with you how to care for their relatives. In fact, some of the family members are troublesome (Participant 8).*

Another added:

*There are some instances where nurses or doctors will give them (relatives) an order; the relatives will rather give the patient drugs from a spiritualist or a herbalist which may lead to contradiction of information which resulting to ineffective therapeutic communication (Participant 13).*

### Environmental barriers

Environmental barriers are obstacles within the environment that inhibit effective therapeutic communication. Almost all nurses and some of the patients asserted that environmental barriers influence therapeutic communication at the ward. Most of the patients expressed how they felt when things they didn’t expect emerged.

Sub-themes that emerged were a noisy environment, new to the hospital environment smell, work overload, mosquitoes, and unconducive environment.

### New to the hospital environment

Adapting to a new environment can be problematic for some people at times, therefore, influencing their ability to communicate effectively.

One Nurse stated;

*You see some of the patients are new to the hospital environment and the mere fact that he or she is in the hospital makes them feel that they are really sick and can’t even communicate with the nurses. They are also already anxious the moment they get to the hospital. This alone can delay therapeutic communication (Participant 11).*

### Noisy environment

Noise can affect any form of communication and in this case therapeutic communication.

The issue of noise is reflected in the following;

*Yeah, some patients put on their radio, some talk on top of their voices and even the nurses chat too much especially, when you call them and they are chatting they do not attend to or mind you because they are concentrating on their talking (Participant 9).*

### Unconducive environment

Communication can be effective only in an environment that is conducive enough for everyone. Participants shared their views on how unconducive the hospital environment is for effective communication. One participant had this to say;

*The mosquitoes disturb us because there are no mosquito nets* etc. *here at night so when you manage to sleep through your own ways and means and the nurses wake us up because time is due for our medications, it becomes a challenge and we need to exercise patience so not to fight or get angry with the nurses that are at the ward (Participant 5).*

*The hospital settings itself is also a barrier. The fact that sometimes the environment is not conducive enough, it may be too warm or cold. Sometimes the patient will tell you that they don’t like the fan whiles others will say that they don’t like the light (Participant 13).*

## Discussions

The study explored the barriers to effective therapeutic communication among patients and nurses in Kumasi. The barriers that were explored include nurses related, patient-related, and environment-related barriers. A key demographic characteristic of patients that were identified as a barrier was age. Similarly, Payne et al., [13], reported that age can serve as a barrier to effective communication. This implies that conditions such as dementia that may set in once you age and causes the level of interpretation and understanding to go down makes it difficult to communicate effectively. Religion was also identified as a barrier as patients who were Muslims would not want females to attend to them but preferred males. People from different parts of Ghana have different cultures and thus patient status, culture, and religion are key barriers to effective therapeutic communication. This implies that religion, age as well as culture has a tendency to influence therapeutic communication. There may be cases where both may belong to the same ethnic group, however, different social orientations and circumstances may affect their communication. Payne et al., [13], also found generational gaps between the elderly and young nurses as key barriers to effective communication. However, culture and ethnic group were not mentioned by Payne et al., [13], but reported by Anderson et al., [14] who emphasized that nurses in interacting with patients from different backgrounds should be sensitive, effective and attach professional attitude.

Language was also identified as a barrier to effective therapeutic communication in this study. Similarly, Quesada [15] reported that in general, the majority of the nurses and patients report that language barrier is an impediment to quality care. The findings corroborate with this study results where patients complained that nurses mostly resort to the Twi language when some of the patients had difficulties in communicating in Twi. Nurses also complained of having patients from different tribes and countries which also makes it difficult for them to communicate effectively.

This study reports that patients complained that they feel like they have been neglected by nurses because they do not promptly attend to them while nurses also complained that, due to the small number of nurses and the workload it becomes difficult attending to all patients as and at when they call. This gives credence to the findings that heavy work schedules of nurses, tough and intensive nursing tasks and the absence of welfare facilities for nurses obstruct communication as reported by Anoosheh et al., [16].

This study reported that several patients complain about their relationship with the nurses and lack of attention. Teutsch [17], reported that nurses undivided attention for patients as they listen to them and observe them gives patients a high level of satisfaction. Interactions with patients therefore eliminate scary thoughts, doubts, and misinterpretations. The researchers do believe that if there is a close relationship between the patient and the nurse, the patient can voice out all their problems to the nurse.

Loghmani, Borhani, and Abbaszadeh [18] in their studies came to the conclusion that nurse-patient communication is declining due to family interference. This gives credence to this current study reporting that family interference is a barrier to effective therapeutic communication. Most of the patients were dissatisfied due to inattention on the part of the nurses and this was a predominant barrier to effective communication in this study. In a similar study, the majority of patients that recounted their experiences on nursing care felt dissatisfied due to neglect [19]. According to McQueen [20], patients in a healthcare facility require information,education, encouragement and support, and nurses are in an ideal position to meet this need.

## Conclusions

Nurse-patient communication is an inseparable part of the patients’ care in every health setting; it is one of the factors that determine the quality of care. However, the results of this study have shown that several factors, which are patient-related, nurse- related and environmental-related pose as barriers to effective therapeutic communication and has ultimately, resulted in reducing effective communication which could affect the quality and comprehensive care delivery at the hospital wards. Authorities at the hospital must ensure that all barriers are eradicated to promote effective therapeutic communication.

## Abbreviation

KATH: Komfo Anokye Teaching Hospital
